# Concomitant use of anti-leishmanial therapy and antibacterial prophylaxis reduces plasma LPS levels and improves several aspects of experimental *Leishmania infantum* infection in golden hamsters

**DOI:** 10.1590/0074-02760240266

**Published:** 2025-09-08

**Authors:** Joanna Reis Santos-Oliveira, Maria Luciana Silva-Freitas, Marcelle da Senhora Cappato, Elaine Marques-Paulo, Milla Bezerra Paiva, Sandra Regina Soares, Dayane Alvarinho de Oliveira, Eduardo José Lopes-Torres, Marcelo Pelajo-Machado, Eduardo Fonseca Pinto, Jose Angelo L Lindoso, Hiro Goto, Alda M Da-Cruz

**Affiliations:** 1 Fundação Oswaldo Cruz-Fiocruz, Instituto Oswaldo Cruz, Laboratório Interdisciplinar de Pesquisas Médicas, Rio de Janeiro, RJ, Brasil; 2 Instituto Federal de Educação, Ciência e Tecnologia do Rio de Janeiro, Núcleo de Ciências Biomédicas Aplicadas, Rio de Janeiro, RJ, Brasil; 3 Instituto Nacional de Ciência e Tecnologia em Neuroimunomodulação, Rio de Janeiro, RJ, Brasil; 4 Fundação Oswaldo Cruz-Fiocruz, Instituto Oswaldo Cruz, Laboratório de Medicina Experimental e Saúde, Rio de Janeiro, RJ, Brasil; 5 Universidade de São Paulo, Instituto de Medicina Tropical de São Paulo, Laboratório de Soroepidemiologia e Imunobiologia, São Paulo, SP, Brasil; 6 Universidade do Estado do Rio de Janeiro, Faculdade de Ciências Médicas, Departamento de Microbiologia, Imunologia e Parasitologia, Rio de Janeiro, RJ, Brasil

**Keywords:** visceral leishmaniasis, bacterial translocation, LPS, golden hamster, immune reconstitution, cellular activation

## Abstract

**BACKGROUND:**

Parasite antigens and plasma lipopolysaccharide (LPS) levels from luminal origin in visceral leishmaniasis (VL) patients are correlated with cellular activation and low CD4^+^T cell counts.

**OBJECTIVES:**

Our aim was to verify whether *Leishmania infantum* infection damages the intestinal barrier and whether combination antimonial/antibiotic contributes to the reduction of LPS levels and immune activation.

**METHODS:**

Golden hamsters were grouped in: G1-uninfected; G2-infected with *L. infantum*; and G3/G4 and G5-infected, treated with antimonial, antibiotic or both drugs, respectively. The treatment initiated 45 days post infection (dpi), daily by 10 days.

**FINDINGS:**

G2, G3, and G4 animals showed a significant increase in spleen weight compared to G1. An elevated parasite load was observed in G2, unlike the G3, G4, and especially, G5, whose decrease was significant at 120 dpi. Intestinal mucosal alterations and elevated LPS levels were observed in G2 group. However, G3, G4 and G5 animals showed lower LPS levels than G2. Moreover, G4 and G5 presented higher CD4^+^T-cell percentages and lower activation levels than G2 and G3, either at 60 or 101-120 dpi.

**MAIN CONCLUSIONS:**

Our results showed evidence of bacterial translocation in experimental VL and that the concomitant use of antimonial and antibiotic may reduce LPS levels, along with an improvement of the immunosuppression and reduction of lymphocyte activation.

Visceral leishmaniasis (VL), also known as kala azar (kala-azar), is caused by *Leishmania infantum* or *Leishmania donovani* and is a serious public health problem associated with poverty that mainly affects tropical and subtropical regions. Its worldwide occurrence ranges from 200,000 to 400,000 new cases per year^1^ ^,^ ^2^ and predominantly affects six countries: India, Bangladesh, Sudan, South Sudan, Ethiopia, and Brazil. In the Americas, more than 90% of the cases occur in Brazil, with approximately 3,000 new cases occurring each year.^3^

Factors related to the initial host immune response, age, environmental conditions, nutritional status, and intrinsic characteristics of the vector influence the clinical presentation, which can range from asymptomatic to systemic immune inflammatory disease.^4^ ^,^ ^5^ Typically, patients with VL experience fever, weight loss, and hepatosplenomegaly. Other signs and symptoms include diarrhoea, vomiting, severe anaemia, pancytopenia, hypergammaglobulinemia and hypoalbuminemia. In general, VL is considered a severe disease that can be fatal if left untreated, and generally the main factors associated with progression to death include spontaneous bleeding and secondary gram-positive bacterial infections.^4^ ^,^ ^6^ ^,^ ^7^

In parallel, VL is characterised by intense lymphocyte depletion resulting from bone marrow impairment and, consequently, reduced generation of T lymphocyte progenitors.^8^ ^,^ ^9^ ^,^ ^10^ Along with this parasite-specific immunosuppression, intense activation of B and T lymphocytes is induced not only by parasitic antigens but also by factors indirectly related to the infection, such as high levels of inflammatory cytokines^4^ ^,^ ^11^ ^,^ ^12^ ^,^ ^13^ and bacterial translocation.^14^ ^,^ ^15^ We demonstrated for the first time that increased levels of lipopolysaccharide (LPS) originating from bacterial translocation were also associated with *Leishmania*-induced immune activation,^14^ as previously described in human immunodeficiency virus (HIV) infection.^16^

The ability of the parasite to infect cells of the gastrointestinal tract,^17^ ^,^ ^18^ along with severe immunosuppression, may contribute to the alteration of intestinal wall permeability and the consequent linkage of gut bacterial products such as LPS. In addition to parasite antigens, villus size augmentation, infiltration of CD4^+^ T lymphocytes and macrophages, and low levels of cytokine expression have also been observed in patients with VL.^18^ Furthermore, the presence of high plasma levels of intestinal fatty acid-binding protein (I-FABP) in the active phase of VL suggests a probable luminal translocation of this LPS^14^ because I-FABP is a cytosolic protein that is only released when the integrity of the enterocyte membrane is disrupted.^19^ In addition, high plasma LPS levels were negatively correlated with the absolute counts of CD4^+^ and CD8^+^ T lymphocytes but seemed to directly influence T lymphocyte activation and the release of pro-inflammatory cytokines such as interleukin (IL)-6, IL-8, and macrophage migration inhibitory factor (MIF). These findings strongly suggest that, in association with *Leishmania* antigens, bacterial translocation products may stimulate the innate and adaptive immune system, creating a chronically activated and inflammatory environment characteristic of VL. This scenario of enhanced immune activation may worsen the clinical condition of these patients by contributing to the exhaustion of the immune response. This phenomenon is characterised by increased phenotypic expression of molecules with inhibitory activity, resulting in functional impairment caused by a decrease in the proliferative capacity and production of interferon (IFN)-γ in response to *L. infantum* antigens,^20^ which can lead to a failure of parasite control.

Regarding experimental VL, most studies on *L. donovani* and/or *L. infantum* infections have used susceptible mice and golden hamsters (*Mesocricetus auratus*). However, the hamster is the most interesting model for progressive VL studies because these animals develop the disease in a very similar way to human VL. Infected golden hamsters present clinical and immunopathogenic characteristics such as asthenia, anaemia, hepatosplenomegaly, hypergammaglobulinemia^21^ ^,^ ^22^ simultaneously with immunosuppression. Curiously, animals are unable to control the parasites despite the presence of IL-2, IFN-γ, and tumour necrosis factor (TNF) in the spleen, liver, and bone marrow.^23^ Indeed, high expression of type I and type II IFN response genes in the spleens of infected Syrian hamsters is not effective in controlling parasite replication and disease.^24^ Furthermore, infected hamsters show a reduction in the proliferative capacity of T lymphocytes against parasitic antigens,^25^ concomitant with intense polyclonal activation of B cells.^26^

In the present study, we took advantage of the similarity of the hamster VL model to human disease, such as gut parasite infection and intense immunosuppression, to investigate intestinal barrier damage and whether antibiotics could reduce bacterial translocation products, such as LPS. Although this translocation of bacterial products does not constitute an infectious process with evident bacteraemia, this mechanism could be an important cofactor for the worsening of the disease, as it may aggravate the cellular activation status of animals. Recently, Lewis et al. induced dysbiosis in hamsters infected with *L. donovani* with broad-spectrum antibiotics and found not only a delay in the onset of the clinical presentation of VL, but also a less severe disease.^27^ This activating effect of bacterial translocation raises the hypothesis that antibiotic therapy could be beneficial as an adjuvant therapy in the human VL.^14^ In the present study, our aim was to investigate whether experimental infection by *L. infantum* can cause intestinal mucosal alterations and bacterial translocation. Moreover, to verify whether antibiotic therapy in combination with anti-*Leishmania* treatment can contribute to the reduction of LPS levels and the degree of cellular activation in *L. infantum*-infected hamsters, which may improve several aspects of experimental VL.

## MATERIALS AND METHODS

*Animals and experimental design* - Outbred adult female Syrian golden hamsters (*Mesocricetus auratus*), six-eight weeks old and weighing 100-120 g, were obtained from the Instituto de Ciência e Tecnologia em Biomodelos, Fundação Oswaldo Cruz (ICTB/FIOCRUZ). Hamsters were housed in a controlled environment at temperature with seasonal lighting conditions (12 h of light and 12 h of darkness), and unrestricted food and water. Their use was approved by the Ethics Committee on Animal Use (CEUA-IOC 032/2015) at FIOCRUZ.

**Fig. 1:**
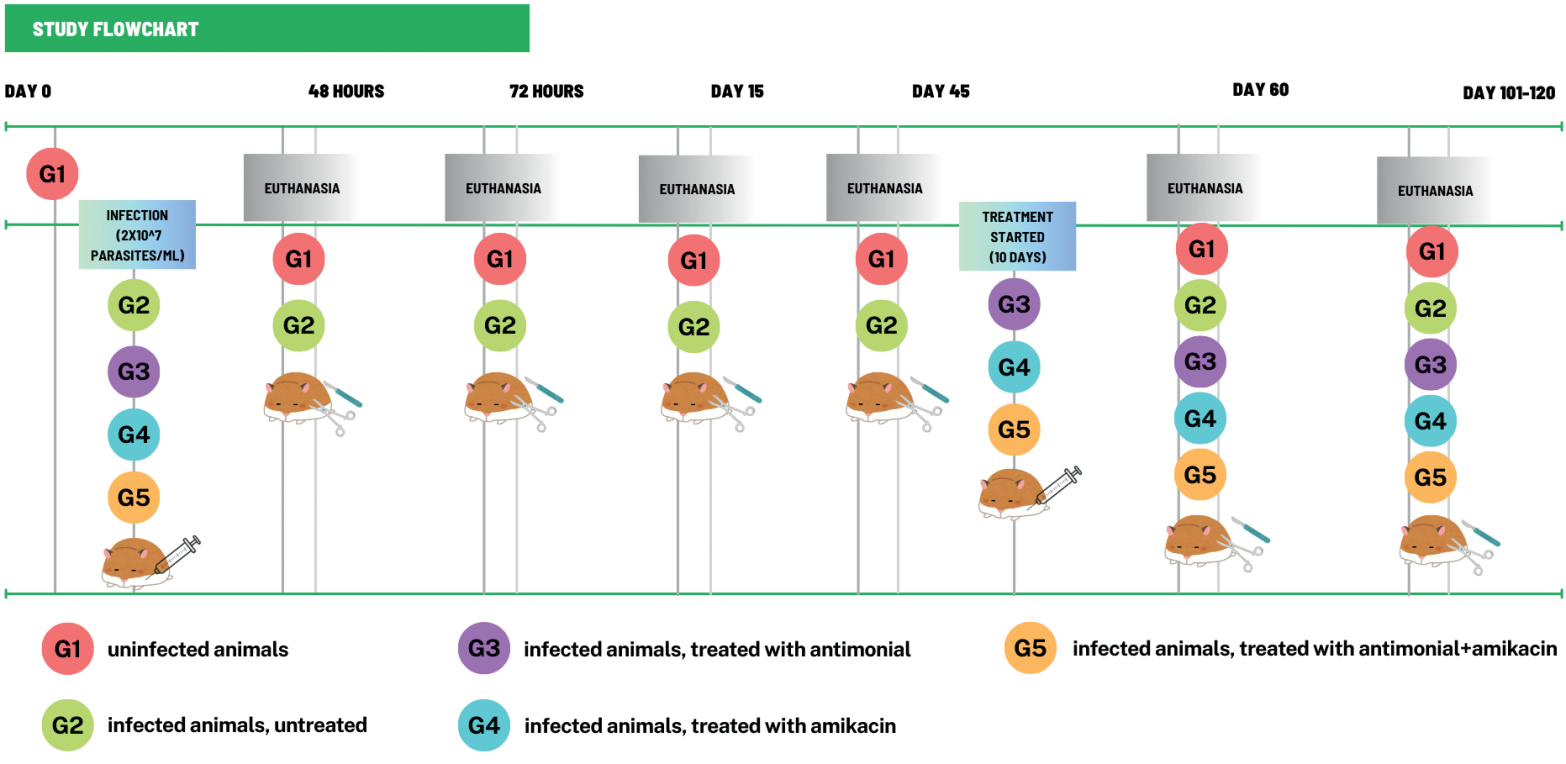
study flowchart. Experimental design of infection, monitoring of disease development and treatment of hamsters infected or not with *Leishmania infantum*. Golden hamsters were infected with 2 × 10^7^ parasites/mL at day 0 and at 45 days post infection (dpi) were treated with meglumine antimoniate, amikacin sulphate or both drugs during 10 days. G1, animals without infection; G2, infected, untreated animals; G3, infected animals treated with anti-*Leishmania* therapy (antimonial); G4, infected animals treated with antibiotic (amikacin); and G5, infected animals treated with both drugs.

The animals were divided into five experimental groups (12 animals per group): G1, animals without infection; G2, infected but untreated animals; G3, infected animals treated with anti-*Leishmania* therapy; G4, infected animals treated with antibiotics; and G5, infected animals treated with both drugs. Two independent experiments were performed. At 45 days post infection (dpi), the animals in each group were treated with the respective drugs. As anti-*Leishmania* treatment, we used meglumine antimoniate 300 mg/mL (N-methylglucamine antimoniate, Glucantime^®^; Sanofi-Aventis, Suzano, SP, Brazil) at a dosage of 100 mg/kg per day. Amikacin sulfate, from the aminoglycoside class, 250 mg/mL (Teuto, Anápolis, GO, Brazil) was used as a broad-spectrum antibiotic at a dosage of 15 mg/kg per day. Drugs were administered intraperitoneally for 10 days. The intervals for obtaining biological material and immunological studies after infection were 48 h, 72 h, 15 dpi, 45 dpi, 60 dpi (15 days after starting treatment), and 101-120 dpi, according to the scheme in Fig. 1.

The hamsters were monitored for appearance, swelling, alopecia, weight loss, skin ulceration, and ascites at all time points. At each time point, the total weight of the animals was measured, and the spleen, liver, small intestine (duodenum), and large intestine (colon) were aseptically removed, weighed, and analysed macroscopically based on surface appearance, colour, and size. Fragments of each organ were stored for further analysis. The critical point for this study was reached approximately four months (101-120 days) after infection.

*Parasites for infection* - *Leishmania infantum* (MHOM/BR/72/strain 46) was provided by Dr Lindoso from Universidade de São Paulo (USP), São Paulo, Brazil. The virulence of the parasite was maintained by continuous passage in golden hamsters through intraperitoneal inoculation of amastigote-rich spleen homogenates. After two months of infection, the amastigotes were isolated from spleen according to the Dwyer protocol^28^ and adjusted to 2 × 10^7^ parasites/mL in a final volume of 100 μL of RPMI 1640 medium for the intraperitoneal infection of the study animals.

*Parasite load determination* - To quantify parasite numbers, apposition (imprint) smears of spleen fragments from infected animals were prepared. The slides were then stained with Panotype (Giemsa) using a commercial Instant Prov kit (NewProv, Pinhais, PR, Brazil), following the manufacturer’s instructions. The imprints were analysed quantitatively under an optical microscope according to the method described by Stauber,^29^ in which up to 1000 amastigotes or cells were counted per slide. The parasite load in the spleen was calculated using the following formula: number of parasites × spleen weight (g) × 2 × 10^4^/number of cells. The results are expressed individually as the number of parasites/g of tissue.

*Evaluation of intestinal parasitism by indirect immunofluorescence* - Immunofluorescence assay for detection of *Leishmania* antigens in colonic tissue was performed. For this, paraffin-embedded colonic sections from infected hamsters were processed for indirect immunofluorescence using a pool of heterologous polyclonal serum obtained from mice experimentally infected with *L. infantum*, at a 1:1000 dilution. The detection was completed using a goat anti-mouse IgG secondary antibody conjugated to Alexa Fluor^®^ 488 (ThermoFisher). Nuclear counter staining was performed using DAPI, and Evans Blue was applied for background suppression. Slides were mounted with ProLong™ Gold Antifade Mountant (ThermoFisher) to preserve fluorescence. Image acquisition was conducted using LSM 710 and/or LSM 980 Airyscan2 confocal microscopes (Zeiss). Negative controls were performed by omitting the primary antibody and substituting it with phosphate-buffered saline (PBS, pH 7.6), while maintaining all other experimental conditions identical to the test protocol, in order to validate the specificity of the immunolabelling.

*Morphometric and histological evaluation of the intestine* - Intestinal tissue samples were collected from the G1 (n = 2) and G2 (n = 4) groups 120 dpi for histological examination. Structural alterations were evaluated in the mucosal, submucosal, and muscle layers of the small intestine (duodenum) and large intestine (colon). The duodenal and colon fragments were fixed in 4% formaldehyde (pH 7.4) for 24 h and then transferred to fresh 4% formalin. The tissues were dehydrated in a graded ethanol series (30% absolute), diaphonised with xylene (Merck, Darmstadt, GER), and embedded in paraffin (Merck). Sections of the tissue, 5 μm in thickness, were then obtained and stained using hematoxylin-eosin and Giemsa (Merck).

Morphometric analyses were performed using ImageJ software (version 1.53 K; Java 1.8.0_172, USA) and images were obtained using an Olympus BX 53 microscope equipped with an Olympus SC100 digital camera. Images were captured with a 40× objective, using the Olympus Cell Sens Entry 1.18 program (Build 16686). Random measurements of the three layers of the duodenum and colon (mucosa, submucosa, and muscular layer) were taken in five distinct areas for each layer from four animals from the infected group and in 10 different areas from two uninfected animals.

*Identification of bacteria in intestinal tissue* - Intestinal tissue samples were collected from the G1 (n = 2) and G2 (n = 4) groups 120 dpi for fluorescence *in situ* hybridisation (FISH). For FISH, the colon fragments were collected and fixed in 4% buffered formalin for 48 h. Subsequently, this tissue was incubated in 10% and 30% sucrose for 24 h at 4ºC, posteriorly embedded in OCT gel (Tissue-Tek), and frozen in liquid nitrogen. Thin sections (5 μm) were recovered on slides prepared with poly-L-lysine (Merck) using a cryostat (Leica CM1850). FISH was performed on each slide using the conditions and buffers described previously,^30^ with 30% formamide in the hybridisation buffer and probes for eubacteria EUB338; EUB338 II; EUB338 III, and NONEUB (control) (Thermo-Fisher). The slides were then stained with DAPI (4′,6-diamidino-2-phenylindole) at a concentration of 0.1 g/mL for 10 min, coated with N-propyl gallate (Merck), and observed with a Zeiss microscope equipped with an AxioImager AxioCam RMC (Zeiss, Germany) using Dye Alexa Fluor 488 filters and the Dye DAPI filter.

*Assessment of plasma LPS levels* - Plasma from heparinised blood was used for LPS quantification. The assays were performed using a commercial kit (Limulus Amebocyte Lysate QCL-1000; Cambrex, Milan, Italy) according to the manufacturer’s instructions. Results were expressed in pg/mL, and the minimum detection limit and sensitivity level of the test was 10 pg/mL.

*Determination of serum anti-Leishmania immunoglobulin levels* - Serum was collected at all previously defined intervals for the determination of anti*-Leishmania* total IgG levels using an enzyme-linked immunosorbent assay. A soluble antigen of *L. infantum* [reference strain IOC/L0579 (MHOM/BR/1974/PP75)] was used, and serum samples were diluted 1:400 in duplicate. Horseradish peroxidase-labelled goat anti-Syrian hamster IgG (Santa Cruz Biotechnology, Inc., Santa Cruz, CA, USA) was diluted to 1:5000 and used as the detection system. The substrate solution consisted of orthophenylenediamine (Merck/Sigma-Aldrich) with 5 μL of 30% hydrogen peroxide (H_2_O_2_), and the colorimetric reaction was stopped with the addition of HCl at 2N. Absorbance was measured at 490 nm using a benchmark microplate reader (Bio-Rad Laboratories, Hercules, CA, USA) and expressed as optical density.

*Immunophenotyping of PBMCs* - PBMCs were obtained by centrifugation in Ficoll-Hypaque (Histopaque-1119, Merck/Sigma-Aldrich). Surface staining was performed after the cell density was adjusted to 3 × 10^5^ cells per tube in 100 µL of 0.1% PBS with azide (Merck/Sigma-Aldrich). To assess the percentages of CD4^+^ T cells and activated cells, PE-Cy5 rat anti-mouse (clone H129.19) and CD25 PE rat anti-mouse (clone 3C7) monoclonal antibodies were used respectively. Both were purchased from BD Biosciences (Franklin, NJ, USA). After incubation, the cells were washed and fixed with 1% paraformaldehyde (Sigma Aldrich, USA) and kept in dark at 4ºC until acquisition by the flow cytometer. At least 20,000 events in the lymphocyte gate were acquired on a FACSCalibur and analysed with CellQuest™ software (BD Biosciences). The analysis region was established by defining the lymphocyte electronic region using forward scatter versus side scatter dot plots. Subsequently, a histogram was created to determine the percentage of CD4^+^ T lymphocytes in the previous region. A new histogram was created to define the percentage of cells expressing CD25 within the CD4^+^ T cell region.

*Statistical analysis* - Results were analysed using GraphPad Software (version 9.0, San Diego, CA, USA) and were expressed as median and interquartile range (IQR: 25-75%). Comparisons were made between the animal groups (G1-G5) at given times in the experimental protocol. For comparing two groups (G1 and G2), the Mann-Whitney test (non-parametric, unpaired) was used. When the five groups (G1-G5) were compared simultaneously, the non-parametric Kruskal-Wallis test (ANOVA) was used. In this case, the Dunns post-test and the false discovery rate (two-stage linear step-up procedure of Benjamini, Krieger and Yekutieli) were used as tests for multiple comparisons. For correlation analyses, non-parametric Spearman correlation was performed. Values of p ≤ 0.05 were considered significant.

## RESULTS

*Natural course of infection in hamsters infected with L. infantum* - Regarding the total weight of the animals, no statistical difference was observed when all groups were compared (Table). However, a significant decrease in total weight was observed in the infected, untreated group (G2) and infected treated with antibiotic group (G4) compared to the uninfected animals (G1) at 60 dpi. At 101-120 dpi, a significant increase in the weight of infected animals, treated with both drugs (G5) was observed compared to that of G4 animals (Table).

**TABLE:** Parasite load and clinical parameters from uninfected hamsters and infected with *Leishmania infantum* treated or not with different scheme therapeutics, at 60 and 101-120 days post-infection (dpi)

		60 dpi				101-120 dpi			
Groups	Outcome	Parasite load (number of parasites/ x10^4^ mg of tissue) (IQR)	Body weight (g) (IQR)	Spleen weight^*a*^ (x10^-3^ g) (IQR)	Liver weight^*b*^ (x10^-3^ g) (IQR)	Parasite load (number of parasites/ x10^4^ mg of tissue) (IQR)	Body weight (g) (IQR)	Spleen weight^*c*^ (x10^-3^ g) (IQR)	Liver weight^*d*^ (x10^-3^ g) (IQR)
G1	Uninfected	NA	157 (145-170)	1.4 (0.9-1.8)	42.0 (39-62.6)	NA	155 (147-166)	0.9 (0.7-1.9)	36.0 (33.0-55.6)
G2	Infected, untreated	2.3 (1.0-108.2)	145 (131-155)	2.9 (1.7-3.4)	39.0 (31.6-59.4)	16.0 (0.6-469.4)	154 (142-172)	3.1 (1.2-4.3)	50.0 (39.4-77.7)
G3	Infected treated with Antimonial	3.0 (1.2-6.0)	145 (135-165)	1.7 (1.2-3.9)	50.0 (37.8-60.0)	1.6 (0.2-11.9)	160 (139-171)	3.0 (1.6-5.1)	48.0 (41.0-73.6)
G4	Infected treated with amikacin	3.4 (0.8-5.2)	144 (129-155)	2.0 (1.0-7.2)	50.0 (38.0-66.4)	1.2 (0.1-11.8)	146 (135-157)	2.4 (1.4-5.0)	42.0 (34.8-58.5)
G5	Infected treated with Antimonial + Amikacin	1.6 (0.5-3.7)	141 (135-160)	2.0 (1.2-4.4)	45.5 (36.6-62.5)	1.4 (0.07-6.5)	160 (150-174)	1.4 (1.0-5.9)	54.0 (36.1-62.9)
p valor (Kruskal-Wallis test)^*^ Dunn’s multiple comparisons test		0.40	0.12	0.34	0.28	0.11	0.32	p < 0.05 G1 vs G3, p < 0.05	0.24
Significant Difference (Mann-Whitney test)^**^		*-*	G1 vs G2, G1 vs G4, p < 0.05	G1 vs G2, p < 0.005	-	G2 vs G3, G2 vs G5, p<0.05	G5 vs G4 p < 0.05	G1 vs G2, G1 vs G3, G1 vs G4, p < 0.05	-
		^*a vs b*^ r = 0.614; p < 0.0005 **(Spearman correlation)**				^*c vs d*^ r = 0.493; p < 0.0005 **(Spearman correlation)**			

The results are expressed as median and IQR: interquartile range; g: gram; NA: not applicable; NS: not significance. ^*^comparison of all groups simultaneously at 60 dpi and 101-120 dpi; ^**^comparison of two groups with each other at 60 dpi and 101-120 dpi.

**Fig. 2:**
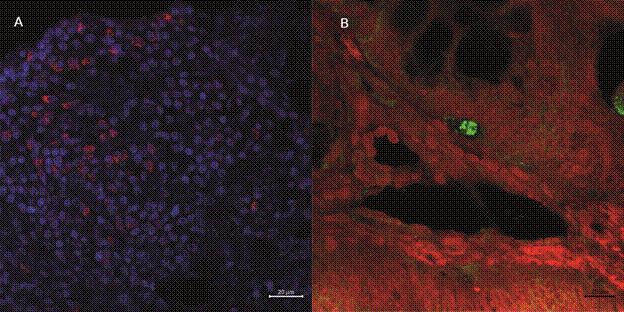
immunofluorescence detection of *Leishmania infantum* amastigotes in colonic tissue from experimentally infected hamsters. (A) Negative control showing absence of fluorescence in colonic tissue from an infected animal processed without the primary antibody. Presence of mucosal and crypt epithelial cells interspersed with a dense eosinophilic infiltrate. (B) Cluster of amastigotes within a perivascular region, surrounded by connective tissue and colonic crypts. Staining was performed using in-house mouse serum from *L. infantum*-infected animals, followed by an Alexa Fluor^®^ 488-conjugated secondary antibody. Host cell nuclei were counterstained with DAPI (blue) and Evans blue (red). Scale bars: as indicated.

The *L. infantum-*infected, untreated animals (G2) showed a significant increase in spleen weight from 45 dpi until the end of the follow up (p < 0.05) compared to the uninfected group (G1) [Table and Supplementary data (Table)]. At 101-120 dpi, animals infected and treated with antimonial (G3) or amikacin (G4) had significantly higher spleen weights than those in the G1 group (p < 0.05; Table). Interestingly, spleen weight in G5 animals did not differ from that in G1 animals at 101-120 dpi. Liver weight was higher in hamsters infected with *L. infantum* (G2, G3, G4, and G5) than in G1 hamsters, although no statistical significance was observed between these groups at 101-120 dpi (Table). A positive correlation was found between liver and spleen weights at 60 and 101-120 dpi [Table and Supplementary data (Fig. 2)].

The parasite load was similar in groups G2, G3, G4, and G5 at 60 dpi (Table). However, at 101-120 dpi, a decrease in parasite load was observed in animals treated with an antimonial (G3) and both drugs (G5) compared to untreated animals (G2) (Table), suggesting that anti-*Leishmania* therapy contributed to parasite control. Although there was no significant difference in relation to G2, the group treated with antibiotic (G4) also presented a drop in parasite load, similar to G3 and G5.

*Evidence of intestinal parasitism in infected hamsters* - To confirm the hypothesis of intestinal involvement during chronic *L. infantum* infection, colonic sections from infected hamsters were evaluated by indirect immunofluorescence reaction. No specific fluorescence staining was observed in the negative controls, confirming the specificity of the immunofluorescent labelling (Fig. 2A). Interesting, a discrete cluster of fluorescent structures was observed in a perivascular region, within the lamina propria, and near colonic crypts, suggesting focally concentrated amastigotes (Fig. 2B). These findings provide direct evidence of discrete mucosal parasitism in the intestinal tract and reinforce histological observations.

**Fig. 3:**
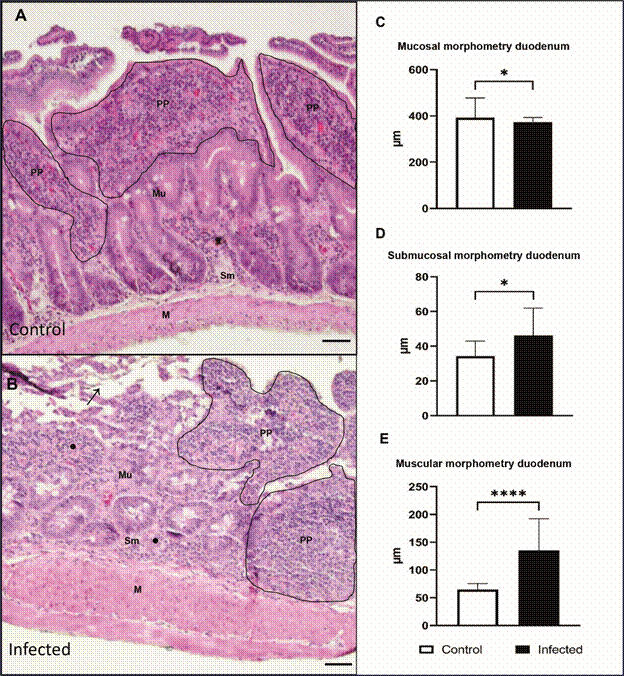
histological section of the duodenum stained with Haematoxylin and Eosin. (A) Duodenum of uninfected hamster (G1) showing the mucosa, submucosa, and muscular layer. (B) Infected golden hamster with *Leishmania infantum* (G2) showing the damaged mucosal surface (black arrow), inflammatory cell infiltrate in the submucosa (black point), and a thickened muscle layer. (C) Mucosal morphometry; (D) Submucosal morphometry; (E) Morphometry of the muscular layer. Representative image of the duodenum of an uninfected (G1, n = 2) and an infected animal (G2, n = 4). Mu: mucosa; Sm: submucosa; M: muscular layer; PP: peyer’s patches; ●: inflammatory infiltrate. Scale bar: 50µm.

**Fig. 4:**
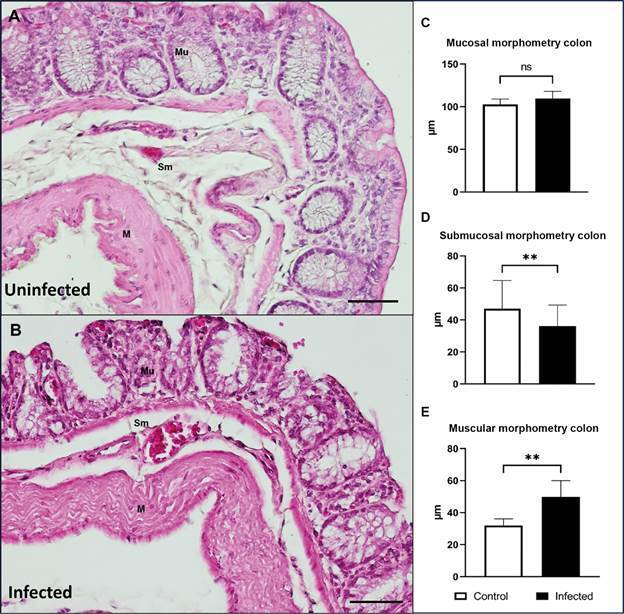
histologic evaluation of the colon stained with Haematoxylin and Eosin. (A) Colon of uninfected hamster (G1), showing intact mucosa, submucosa and muscular layer. (B) Colon of infected hamster golden hamster with *Leishmania infantum* (G2), exhibiting histological variations in shape and length of the crypts, damage to the striated border of the colon mucosa, a narrow submucosa, and a thick muscular layer. (C) Mucosal morphometry; (D) Submucosal morphometry; (E) Morphometry of the muscular layer. Representative image of the colon of an uninfected (G1, n = 2) and an infected animal (G2, n = 4). Mu: mucosa; Sm: submucosa; M: muscular layer. Scale bar: 50µm.

*Disorganisation of the intestinal epithelium and bacterial translocation in infected hamsters* - In order to evaluate the alterations caused by *L. infantum* infection in the intestine, histopathological and morphometric analyses of the duodenum and colon of the animals were performed. A comparison of the G1 and G2 groups revealed a normal appearance of the three layers of the duodenum and colon in the uninfected group, whereas structural alterations were observed in the tissues of chronically infected hamsters (Figs 3-4).

The duodenal mucosal layer was intact in uninfected animals (G1). However, infected hamsters (G2) exhibited structural damage to the mucosal surface, thickening of the submucosa accompanied by inflammatory cell infiltration, and a visibly thick muscular layer (Fig. 3A-B). In the colon, ruptures were observed in the crypts of Lieberkühn. The colonic mucosal surface was damaged at the striated border. Additionally, we observed thinning of the submucosa with a few inflammatory cells and a robust muscular layer (Fig. 4A-B). Morphometric analysis of the duodenum revealed a thin mucosal layer (372.7 µm, IQR: 250.6-393.0 µm) and thickening of the submucosa (46.15 µm, IQR: 30.03-61.98 µm) and muscularis (135.6 µm, IQR: 104.0-192.4 µm) layers in the infected hamster compared to the thickness of the mucosa (392.40 µm, IQR: 345.80-482.10 µm), submucosa (34.31 µm, IQR: 23.31-42.98 µm), and muscularis (64.44 µm, IQR: 57.70-75.95 µm) layers in the uninfected animals (Fig. 3C-E). Despite the striated border alteration, morphometric analyses of the colon did not show a significant difference in the mucosa between infected hamsters (109.60 µm, IQR: 100.1-118.80 µm) and uninfected hamsters (102.70 µm, IQR: 97.40-109.40 µm). We also observed a thinner submucosa (36.15 µm, IQR: 28.65-49.38 µm) and a significantly thickened muscularis (49.90 µm, IQR: 35.78-60.30 µm) in the infected animals compared to the thickness of the submucosa (47.05 µm, IQR: 41.88-65.68 µm) and muscularis (31.95 µm, IQR: 27.25-45.93 µm) in the uninfected animals (Fig. 4C-E).

**Fig. 5:**
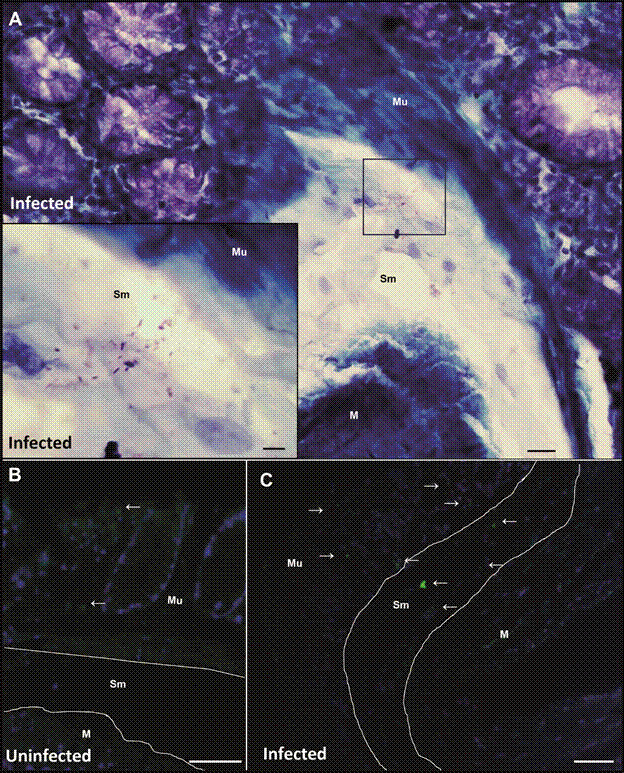
evidence of bacterial translocation in large intestine of golden hamsters infected with *Leishmania infantum*. (A) Histological sections of large intestine (colon) stained with Giemsa and (B and C) Fluorescence microscopy using DAPI and fluorescence *in situ* hybridisation (FISH) for analysis of bacterial invasion in the submucosa layer. (A) Infected-hamster tissue with *L. infantum* showing mucosa, submucosa, and muscular layer, with bacteria in the submucosa. Detail of the showing cocci-shaped and bacilli bacteria in the submucosa. (B and C) Fluorescence microscopy showing bacteria (green) and host tissue (DAPI). (B) Sections of colon from uninfected hamster with absent bacteria. (C) *Leishmania*-infected tissue showing bacteria in the mucosa, and invasion of bacteria in the submucosa. Mu: mucosa; Sm: submucosa; M: muscularis; arrow, bacteria. Scale bar: A - 20µm; detail of A - 10µm; B and C - 50µm.

Further histological examination of the intestinal tissue of the colon from the infected hamsters showed bacteria invading the intestinal submucosa. Different experiments using bright-field and fluorescence microscopy were performed to identify bacterial translocation. Giemsa staining revealed the presence of cocci and bacilli in the colonic submucosa of infected animals (Fig. 5A). The invasive process was confirmed by FISH experiments showing that in samples from infected hamsters, bacteria (green) were identified not only in the crypts of Lieberkühn but also inside the submucosa (Fig. 5C), suggesting that damage to the epithelial layer in the mucosa promotes bacterial invasion into the submucosa. In the colon of uninfected animals, bacteria were present only on the surface of the mucosal layer and were localized in the intestinal lumen and crypts of Lieberkühn, as expected (Fig. 5B).

**Fig. 6:**
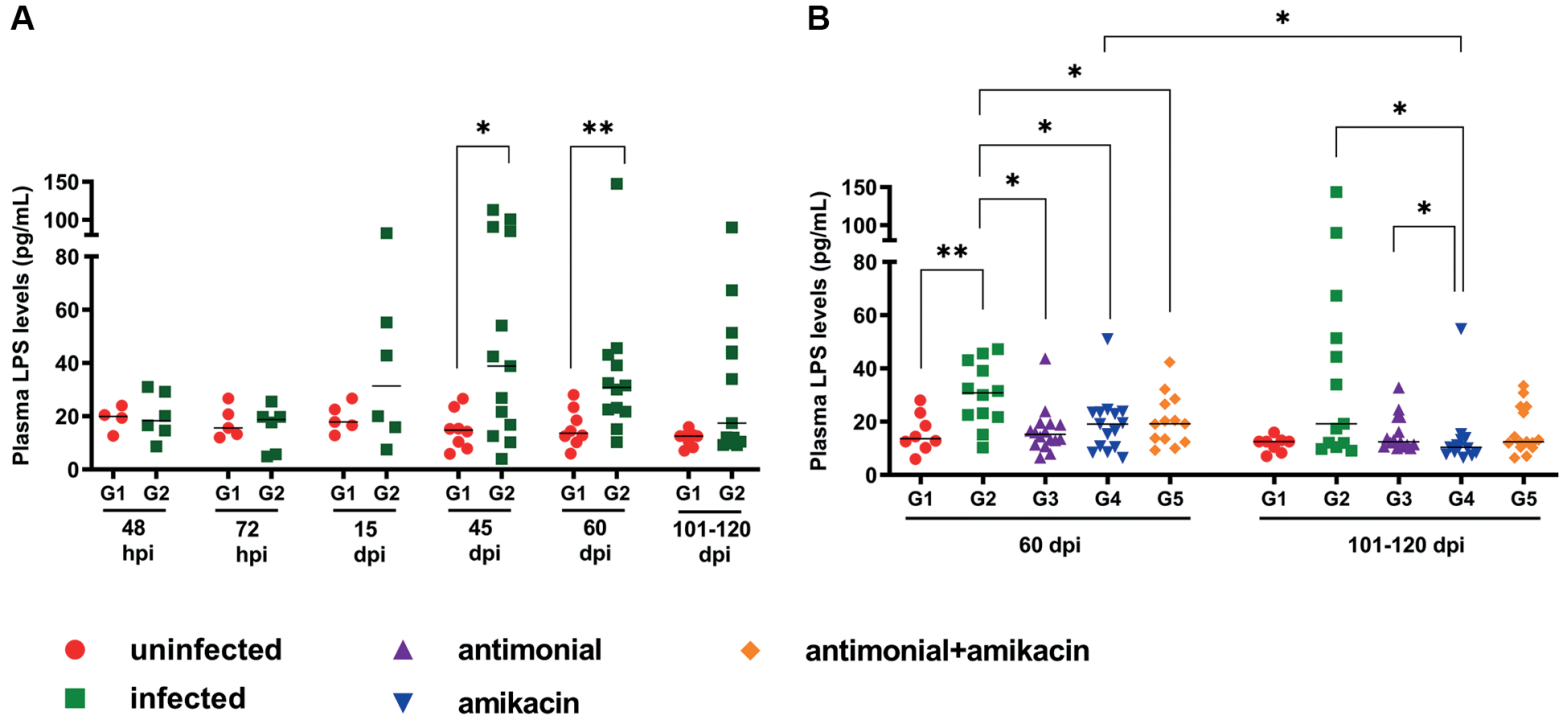
prospective evaluation of plasma lipopolysaccharide (LPS) levels in golden hamsters infected with *Leishmania infantum*. (A) Assessment of LPS levels in uninfected hamsters (G1) and infected-untreated animals (G2) that were followed over 101-120 days post-infection (dpi) and (B) in all experimental groups followed at 60 and 101-120 days post-infection. At 45 days post-infection, the treatment of the animals was started with the respective drugs of each group. G1: uninfected animal (red circle), G2: infected and untreated (green square), G3, G4 and G5: infected and treated with antimonial (purple triangle), amikacin (blue inverted triangle) or both drugs (orange diamond), respectively. Each point in the figure represents an animal, and the horizontal bar represents the median of the values. ^*^p < 0.05 [t test, non-parametric, Mann-Whitney test or analysis of variance (ANOVA), non-parametric, Kruskal-Wallis test ].

*Anti-Leishmania and antibiotic therapy decrease LPS levels in L. infantum-infected golden hamsters* - Previous results from our group showed that LPS levels were elevated in the active phase of human VL in association with the activation status and decreased CD4^+^ T cell counts.^14^ In comparison to the control group (G1), an increase in plasma LPS levels was observed in the infected, untreated animals (G2) from 15 dpi until the end of the observation period, although this was not significant at 101-120 dpi [Fig. 6A and Supplementary data (Table)].

LPS levels at 60 dpi were significantly lower in all treated groups (G3, G4, and G5) than in the untreated, infected group (G2) (Fig. 6B). The same profile was observed at 101-120 dpi, especially for G4, in which LPS levels were significantly lower than those in G2 (Fig. 6B). Interestingly, no difference was observed between the infected and treated groups (G3, G4, and G5) at 60 dpi; however, at 101-120 dpi, it was possible to verify that the animals treated with antibiotic (G4) had significantly lower LPS levels than the G3 animals.

**Fig. 7:**
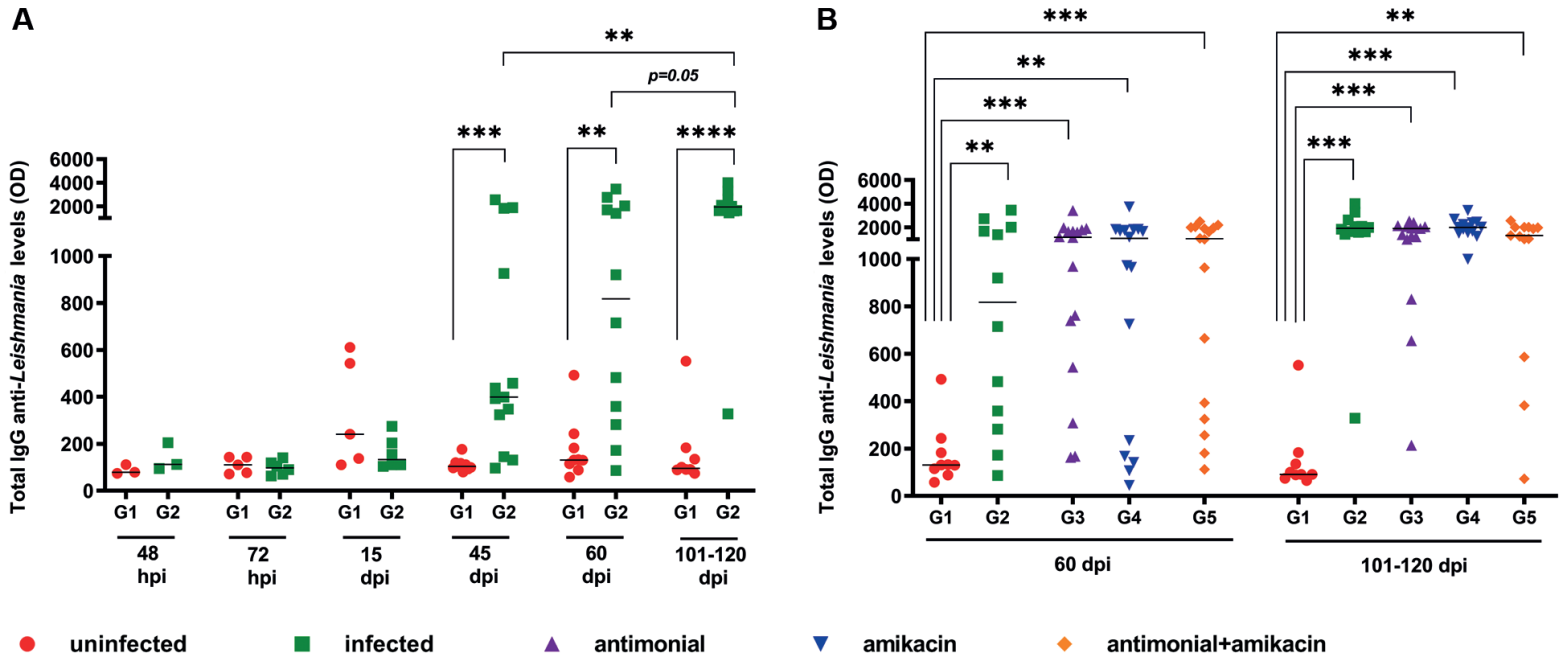
prospective evaluation of total IgG anti-*Leishmania* levels in golden hamsters infected with *Leishmania infantum*. (A) IgG levels were measured by enzyme-linked immunosorbent assay (ELISA) and performed in unifected (G1) and infected-untreated animals (G2) that were followed over 101-120 days post-infection (dpi) and (B) in all experimental groups followed at 60 and 101-120 days post-infection. At 45 days post-infection, the treatment of the animals was started with the respective drugs of each group. G1 (red cicle), G2: infected and untreated (green square), G3, G4 and G5: infected and treated with antimonial (purple triangle), amikacin (blue inverted triangle) or both drugs (orange diamond), respectively. Each point in the figure represents an animal, and the horizontal bar represents the median of the values. ^*^p < 0.05 [Analysis of variance (ANOVA), non-parametric, Kruskal-Wallis test ].

*High levels of anti-Leishmania IgG in infected hamsters regardless of antimonial and antibiotic treatment* - As the anti-*L. infantum* IgG levels are associated with parasite persistence in leishmaniasis,^31^ ^,^ ^32^ we evaluated the impact of antimonial and antibiotic therapy on this parameter. The anti-*L. infantum* IgG levels remained low at the beginning of follow-up, regardless of the presence of VL. However, at 45 dpi, a significant increase in total IgG levels (p < 0.05) was observed in the G2 group compared to the uninfected group (G1) [Fig. 7A and Supplementary data (Table)]. This profile was maintained until the end of follow-up.

In addition, it was found that all groups that received treatment with the proposed drugs (G3, G4, and G5) had significantly higher titres (p < 0.05) of anti-*L. infantum* IgG than those in G1 at 60 and 101-120 dpi, but no difference was found between the treated groups (Fig. 7B). Also, the anti-*Leishmania* IgG levels were positively correlated with parasite load (r = 0.31; p < 0.05) and spleen weight (r = 0.33; p < 0.05) in all infected groups evaluated at 101-120 dpi [Supplementary data (Figs 1-2)]. These results reinforce the idea that the maintenance of IgG levels may be associated with the presence of the parasite.

*Antibiotic and antimonial therapy improved the immune impairment of L. infantum-infected animals* - Previous studies have shown that the active phase of VL is characterised by intense immune impairment, including low CD4^+^ T lymphocyte counts. In addition to immunosuppression, intense cell activation has been observed in human VL. In this context, experimentally infected hamsters were evaluated for the percentage of CD4^+^ T cells and the degree of T cell activation by flow cytometry.

**Fig. 8:**
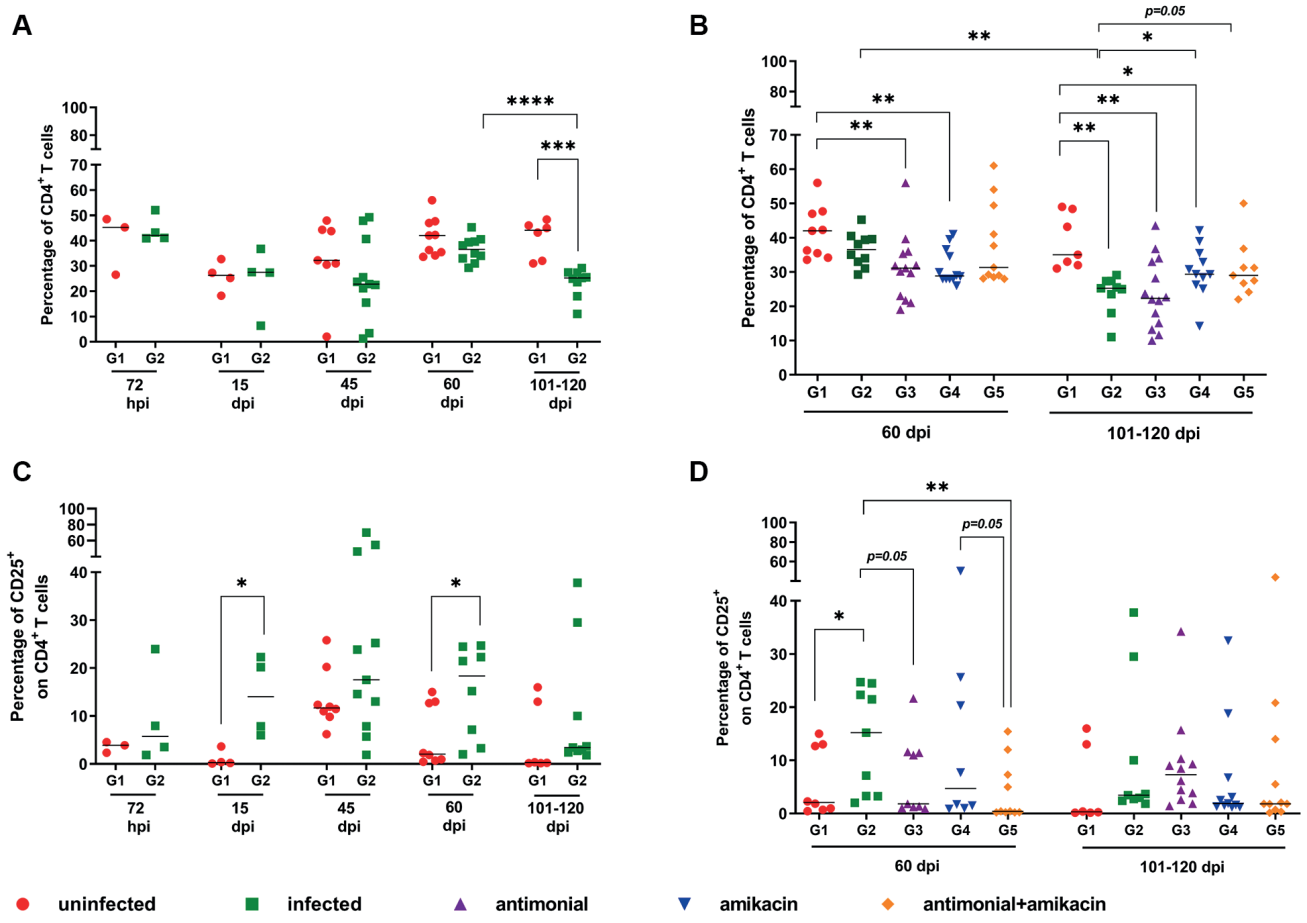
prospective evaluation of the CD4 T-cell levels (A and C) and cellular activation degree (B and D) in golden hamsters infected with *Leishmania (L.) infantum*. The percentages of CD4^+^ in PBMC (A and C) and CD25^+^ on T CD4^+^ lymphocytes (B and D) was obtained by flow cytometry and were evaluated in unifected (G1) and *Leishmania* infected-untreated animals (G2) that were followed of 72 hours up to 101-120 days post-infection (dpi) (A-B). The same evaluation was performed in all experimental groups that followed at 60 and 101-120 dpi (C-D). At 45 dpi, the treatment of the animals was started with the respective drugs of each group. G1: uninfected (red circle), G2: infected and untreated (green square), G3, G4 and G5: infected and treated with antimonial (purple triangle), amikacin (blue inverted triangle) or both drugs (orange diamond), respectively. Each point in the figure represents an animal, and the horizontal bar represents the median of the values. ^*^p < 0.05 [t test, non-parametric, Mann-Whitney test or analysis of variance (ANOVA), non-parametric, Kruskal-Wallis test ]. Sample acquisition was performed using the FACSCalibur cytometer and analysis was performed using the CellQuest™ software.

A decrease in the percentages of CD4^+^ T cells was observed from 45 dpi in the G2 group, but this was significant (p < 0.01) only at 101-120 dpi when compared to G1. This decrease was also significant in the G2 group at 101-120 dpi when compared to 60 dpi (Fig. 8A).

Interestingly, lower percentages of CD4^+^ T cells were observed in infected animals, regardless of anti-*Leishmania* treatment (G3) when compared to G1 at both 60 and 101-120 dpi (p < 0.05). At 101-120 dpi, animals treated with antibiotics (G4) and both drugs (G5) showed a significant increase (p < 0.05) in the percentages of CD4^+^ T lymphocytes compared to the untreated infected group (G2) (Fig. 8C). Although these percentages were still lower in the treated groups (G4 and G5) than in the uninfected group (G1) at this time, these results suggest that treatment contributed to a slight recovery of this lymphocyte subpopulation (Fig. 8C). In addition, G5 showed no difference in this parameter when compared to the uninfected group (G1), unlike G4 that remained with significantly smaller percentages.

Finally, we analysed the percentage of cells expressing CD25, which is associated with cellular activation, within the population of CD4^+^ T lymphocytes. At all times analysed, the infected group (G2) had higher levels of activation than the uninfected group (G1) [Fig. 8B and Supplementary data (Table)]. However, this increase was significant only between G1 and G2 at 15 dpi (p < 0.05) and 60 dpi (p < 0.01).

The animals treated with antimonial alone (G3) and those treated with both drugs (G5) had significantly lower activation levels than infected, untreated animals (G2) at 60 dpi, similar to uninfected animals (G1) (Fig. 8D). Although not significant, the G5 animals showed the lowest degree of activation compared to the other infected groups, treated or not, at 60 (p = 0.05, compared to G4) and 101-120 dpi (Fig. 8D).

It is noteworthy that, we observed a significant positive correlation between the spleen weight and LPS levels (60 dpi: r = 0.25; 101-120 dpi: r = 0.40; p < 0.05) and percentages of CD25^+^ T cells (60 dpi: r = 0.61; 101-120 dpi: r = 0.36; p < 0.05) [Supplementary data (Fig. 2)]. Although it was not significant, LPS levels also correlated positively with percentages of CD25^+^ in CD4 T cells (r = 0.26; p = 0.05) at 101-120 dpi [Supplementary data (Fig. 2)]. In contrast, a negative correlation was observed between levels of CD25^+^ in CD4 T cells and CD4^+^ T cell percentages also at 101-120 dpi (r = -0.37; p < 0.05) [Supplementary data (Fig. 2)]. These results may suggest the impact of cellular activation on deficient immune recovery in VL as well as the relationship between this parameter and microbial translocation.

## DISCUSSION

Components of the *L. infantum* parasite are involved in the immunological abnormalities observed in VL, whether through suppressive or activating mechanisms. In addition, we have provided evidence for bacterial translocation in human VL, implicating these products as possible cofactors for the immune inflammatory activation status of patients with VL and consequent impaired T cell function.^14^ ^,^ ^33^ Elevated levels of I-FABP indicate mucosal barrier breakdown, whereas augmentation of LPS and soluble CD14 indicates the presence of gram-negative bacterial products in circulation.^34^ ^,^ ^35^ These findings prompted us to assess the possibility of bacterial translocation from the damaged mucosa and to evaluate whether antibiotic therapy in addition to anti-*Leishmania* treatment improves the clinical and immunological aspects of the disease. The *L. infantum*-infected hamster VL model was chosen to prospectively evaluate the effects of pentavalent antimonial (G3), amikacin (G4), or both (G5) on disease outcome.

Similar to what has been described in the literature regarding intestinal parasitism in human VL,^18^ ^,^ ^36^ and the morphological changes caused by the parasite in this region,^37^ ^,^ ^38^ ^,^ ^39^ our study also demonstrated the presence of clusters of amastigotes in the perivascular region of the large intestine, surrounded by connective tissue and colonic crypts. This colocalisation of parasites with dense eosinophilic infiltrates, as observed in immunofluorescence studies, corroborates with frequently reported mixed inflammatory infiltrate with marked presence of eosinophils associated with the presence of amastigote forms.^40^ We also showed that the infected animals presented histological and morphometric alterations in terms of intestinal layer atrophy and hypertrophy, which is in accordance with previous study.^41^ Passos et al. also investigated the effects of *L. infantum* infection on the intestinal microbiota of infected hamsters in relation to the genera *Bifidobacterium spp*. and *Lactobacillus* spp., which are well-known probiotics that help maintain intestinal homeostasis.^41^ No differences were found in the relative abundance of these bacterial genera between infected and control animals; however, they were associated with a lower parasite load in the colon of infected animals, which may indicate a protective role of the intestinal tissue, potentially ameliorating the damage caused by the parasite.^41^ Regardless of dysbiosis, it is possible that a high parasite load in the active phase of VL can generate lesions that facilitate bacterial translocation from the mucosal surface to the submucosa, as observed in our study. This may promote the invasion of bacterial products into the circulation and trigger immune response activation mechanisms. In addition, amikacin therapy appeared to have beneficial effects in terms of reducing the parasite load, decreasing LPS levels, suppressing the decrease in CD4^+^ T cell counts, and decreasing the degree of cellular activation.

All *L. infantum* infected, untreated hamsters (G2) evaluated in this study presented some degree of clinical, parasitological, or immunological commitment/alterations related to VL.^14^ ^,^ ^42^ ^,^ ^43^ ^,^ ^44^ Weight loss, spleen and liver enlargement, augmentation of parasite load and IgG levels, decreased CD4^+^ T lymphocyte percentages, and elevated cell activation were consistently observed, although none of the infected hamsters were severely ill. Three conditions could explain this benign clinical evolution: (1) the lower virulence of the *L. infantum* strain used for challenge (MHOM/BR/72/strain 46); (2) the well-nourished condition of the animals;^10^ ^,^ ^45^ and (3) the period of observation (101-120 days), which was not sufficient to observe the consequences of impaired lymphoid organs.^10^ ^,^ ^46^

At the end of the study, the three therapeutic interventions were associated with an improvement in VL outcome, in addition to a striking reduction in parasite load. Interestingly, the parasite load in animals receiving amikacin alone (G4) was comparable to that observed in the antimonial-treated group (G3). This was not surprising because the effects of aminoglycosides such as paromomycin are well reported in VL and tegumentary leishmaniasis.^47^ ^,^ ^48^ ^,^ ^49^ Remarkably, amikacin alone, but not antimonial alone, seems to impact in the reduction of the spleen weight compared with that in G2 at 120 dpi, although without significant difference. Moreover, the combination therapy (G5) resulted in a reduction in parasite load coincident with spleen weight values closest to those of the uninfected animals at 120 dpi. Other authors have reported that treatment of VL hamsters with a cocktail of antibiotics alone results in a delay in total weight loss and spleen enlargement.^27^ Together, these results indicate that antibiotics can interfere with the pathogenesis of VL, and the improvement in the G5 group suggests a probable synergistic effect of these drugs on the parasite, eliminating it more efficiently.

*Leishmania infantum* is capable of infecting intestinal cells in humans^18^ ^,^ ^50^ and in experimental models.^27^ Macroscopic changes in the small intestine, such as ulcerations and inflammation in the rectosigmoid region of a patient with VL,^39^ as well as the presence of a high parasite load in the cecum and colon of dogs infected with this protozoan^51^ have been described. In the present study, the histological alterations in the intestinal tissues of the infected animals (G2) were accompanied by inflammatory cell infiltration in the submucosal layer of the duodenum and intense ruptures in the crypts of Lieberkühn in the colon. According to this finding on mucosal barrier damage in infected animals, *cocci* and *bacilli* in the submucosa of the colon were observed by conventional light microscopy and confirmed by FISH. These results suggest that morphological changes in the intestine can affect the selective permeability of this tissue, which, in turn, favors the passage of pathogens and microbial products.

Bacterial products from bacterial translocation have been implicated in the impairment of effector immune responses in several infectious and non-infectious diseases.^16^ ^,^ ^39^ ^,^ ^40^ LPS from gram-negative bacteria is involved in immunostimulatory function and associated with the presence of several molecules involved in innate and/or adaptive immune activation, either in the systemic circulation^14^ ^,^ ^15^ ^,^ ^40^ or even in the liver.^27^ Increased LPS levels were observed in the precocious phase of infection (15 dpi) in infected, untreated hamsters (G2) and were maintained throughout the observation period. The histopathological changes and the presence of bacteria invading the intestinal submucosa in infected hamsters indicate that this endotoxin may reach the circulation through microbial translocation from the intestinal lumen. Recently, fluorescently labelled dextran translocation along with LPS staining in the livers of infected hamsters suggested that bacterial translocation from the gut is a feature of VL.^27^

It is worth noting that changes in the intestinal microbiome can also contribute to the occurrence of bacterial translocation. Furthermore, certain pathogens can also directly contribute to intestinal dysbiosis by triggering significant inflammatory processes.^52^ In this sense, we believe that the parasitism of intestinal cells along with the intense inflammatory response caused by *L. infantum* infection could be involved in the dysbiosis and anatomical-functional damage to the gastrointestinal barrier, which in turn can promote subsequent translocation of bacterial products into the systemic circulation.

Interestingly, the therapeutic intervention seemed to have had an impact on bacterial translocation, as days after starting antimonial and/or amikacin treatment, LPS levels were lower in all treated groups than in untreated animals (G2). Higher levels of LPS were maintained in G2 group compared to the treated groups, whose suppression of the LPS increase was maintained until the end of the follow-up. This effect on LPS levels observed in antimonial-treated animals is believed to be related to a decrease in parasite load, which could indirectly contribute to less damage to the mucosa, and therefore, a lower degree of translocation. It is noteworthy that the group treated with amikacin alone (G4) presented suppression of LPS augmentation in a similar way to that observed in antimonial-treated animals, showing the importance of this antibiotic in reducing the translocation of bacterial products. Thus, amikacin, in addition to killing intestinal mucosal parasites,^18^ ^,^ ^50^ can also play a beneficial role in reducing intestinal gram-negative bacterial replication and, consequently, bacterial translocation. In turn, this may contribute to a decrease in systemic activation and, consequently, an exhausted immune status, positively influencing the prognosis of VL. At the same time, the concomitant administration of both drugs showed no potentiation of the effect on LPS levels, which could be related to the short evaluation period. Even so, the G5 group was positively affected by the concomitant use of the antibiotic and pentavalent antimonial, as they presented the lowest parasite load among the experimental groups and lower T activation levels.

It is important to note that great variability in LPS levels occurred at all time points and augmentation of this molecule was observed in approximately 50% of the animals. These results indicate that LPS augmentation may be related to the degree of clinical impairment, especially gut damage, spleen enlargement, and cellular activation. In this connection, LPS levels were positively correlated with spleen weight and with the degree of cellular activation. Activation, in turn, was positively correlated with spleen weight. Thus, animals with higher LPS levels would experience more intestinal damage and activation, which could be due to the parasite load.

VL itself is characterized by high levels of T and B lymphocyte activation, hypergammaglobulinemia, and high levels of inflammatory cytokines.^13^ ^,^ ^53^ ^,^ ^54^ *Leishmania* antigens and microbial products account for this inflammatory status. In this study, the levels of total *L. infantum-*reactive IgG progressively increased throughout the infection follow-up period. However, at 101-120 dpi, anti-*Leishmania* IgG levels were elevated in all infected animals, and antimonial therapeutic intervention did not interfere with these titres. The maintenance of high IgG levels may be related to sustained *Leishmania*-antigenic stimuli. Despite the strong reduction in parasite load detected in all three treatments, the parasites persisted in the spleen. Thus, the remaining parasites could sustain B-lymphocyte activation and consequent IgG release.

In addition to systemic immune activation, the depletion of T lymphocytes is a hallmark of human^42^ ^,^ ^55^ and canine VL^56^ immunopathogenesis. Owing to the lack of immunological reagents, such as hamster-specific antibodies against cell surface markers, little is known about T cell phenotype profiles in the VL hamster model. However, it is already known that the depletion of CD4^+^ T cells resulting from immunosuppression has been associated with an increase in parasite burden in golden hamsters^57^ and human VL relapses.^43^ This depletion may be a consequence of CD4^+^ T cells apoptosis in the liver and spleen of *L*. *chagasi*-infected hamsters induced by *Leishmania* antigen stimulation in the early period of infection.^58^ Recently, a drop in lymphocyte cell count was found to be inversely correlated with cortisol levels in a VL hamster model.^59^ Taking advantage of antibody cross-reactivity, we verified that *Leishmania*-infected hamsters exhibited a reduction in the percentage of CD4^+^ T cells. Importantly, these cells are more activated. Regardless of the therapeutic scheme, the infected animals still had a lower percentage of CD4^+^ T cells than the uninfected animals (G1), except for G5 animals that were submitted to combined therapy. Interestingly, at the final time point of observation, the amikacin-treated groups, especially G5 group, presented a higher percentage of CD4^+^ T cells along with lower activation levels than the infected, untreated group (G2), or even treated with antimonial alone (G3). This indicated that amikacin-treated animals were less immunocompromised. The reason the activation levels decreased after the end of antibiotic treatment remains unclear; however, we believe that it may act on the parasite itself^48^ and/or on bacterial translocation (LPS levels). Alternatively, the antibiotic may have had an effect on pathobionts, which, if present, contributed to VL progression.^27^

In addition to the parasite, LPS is another factor that may contribute to immune activation in VL. In this scenario, these products could worsen immune impairment through the depletion of CD4^+^ T cells^60^ in a mechanism similar to that of activation-induced cell death as well as by increasing the degree of cell activation. In infected hamsters, we observed not only that LPS levels correlated positively with lymphocyte activation but also that activated T cell levels correlated negatively with CD4^+^ T cell counts. This reinforces the notion that bacterial products may exert deleterious effects on the effector immune response in these animals, creating a vicious circle.

The outbred animal model is undoubtedly the most interesting for studying clinical aspects and progression of VL, but we observed high intra-group variability in some of parameters evaluated. Moreover, the evaluation of the activating effects of LPS on the effector immune response was limited by scarcity of immunological inputs available for this model. Finally, our sample sizes may have influenced the statistical power of the comparisons between the experimental groups.

**Fig. 9:**
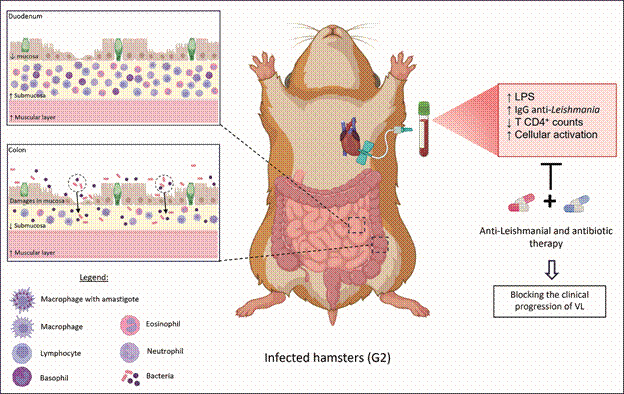
schematic representation of the bacterial translocation in experimental visceral leishmaniasis (VL) and its systemic consequences for the immunopathogenesis of the disease and the impact of combined therapy. The figure represents an animal infected with *Leishmania infantum* presenting intestinal damage (duodenum and colon) resulting from the parasitism and inflammatory response itself. The increase in intestinal permeability culminates in translocation of bacterial products from intestinal lumen to the bloodstream, such as LPS from Gram (-) bacteria, resulting in systemic disturbances such as increased cellular activation, increased levels of anti-*Leishmania* IgG and decreased CD4+ T lymphocyte counts. Our hypothesis is that this scenario can be alleviated by combined anti-*Leishmania* and antibiotic therapy, leading to blocking the clinical progression of VL.

Despite these limitations of the study, the present work provides evidence not only that VL infection is associated with damage to the epithelial layer in the mucosa but also that it can favour bacterial invasion into the submucosa. Moreover, this study showed that treatment with amikacin was associated with an improvement in important parameters of VL, such as parasite load reduction, lower plasma LPS levels, and consequently, activation levels, in addition to improving immune reconstitution. Finally, our data suggest that the therapeutic synergism between pentavalent antimonial and amikacin may improve several aspects of VL infections, especially the cellular activation (Fig. 9). This hypothesis should be investigated in clinical trials of VL in humans.
