# Supplementary material for: Concomitant use of anti-leishmanial therapy and antibacterial prophylaxis reduces plasma LPS levels and improves several aspects of experimental Leishmania infantum infection in golden hamsters
Source: Mem Inst Oswaldo Cruz. 2025 Sep 8;120:e240266. doi: 10.1590/0074-02760240266 (PMC12418786; doi:10.1590/0074-02760240266)
Supplement: Supplementary file 1 [file 1678-8060-mioc-120-e240266-s.pdf]

TABLE

Clinical and immunological parameters of hamster groups uninfected (G1) and infected with *Leishmania (L.) infantum* (G2) followed-up at different time points

| | G1 | G2 | | G1 | G2 | | G1 | G2 | | G1 | G2 | | G1 | G2 | | G1 | G2 | |
|---|---|---|---|---|---|---|---|---|---|---|---|---|---|---|---|---|---|---|
| | 48 hpi (IQR) | | p-value | 72 hpi (IQR) | | p-value | 15 dpi (IQR) | | p-value | 45 dpi (IQR) | | p-value | 60 dpi (IQR) | | p-value | 101-120 dpi (IQR) | | p-value |
| Body weight (g) | 116.6 (108.8-123.6) | 109.2 (106.8-116.8) | NS | 116.8 (100.8-132.9) | 117.6 (108.8-125.9) | NS | 116.4 (115.6-127.8) | 134.6 125.6-137.7) | NS | 135.6 (125.3-157.1) | 143.8 (131.3-146.5) | NS | 157.2 (145.0-170.3) | 145 (131.3-155.0) | **$p < 0.05$** | 155 (147-165.6) | 153.7 (141.6-172.3) | NS |
| Spleen weight (x10$^{-3}$ g) | - | - | NA | - | - | NA | 1.1 (0.9-1.4) | 0.9 (0.8-3.0) | NS | 1.5 (0.9-1.7) | 2.1 (1.2-3.2) | **$p < 0.05$** | 1.6 (1.0-2.0) | 3.0 (1.6-4.0) | **$p < 0.05$** | 1.0 (0.8-2.0) | 3.3 (1.2-4.1) | **$p < 0.05$** |
| Liver weight (x10$^{-3}$ g) | - | - | NA | - | - | NA | 38.1 (37.6-39.0) | 36.3 (31.6-38.8) | NS | 38.7 (33.7-51.5) | 47.4 (36.9-53.2) | NS | 42.2 (39.0-62.6) | 39.2 (31.6-59.4) | NS | 36.0 (33.0-55.6) | 49.8 (39.4-77.7) | NS |
| LPS (pg/mL) | 19.9 (14.4-23.1) | 18.3 (13.1-29.6) | NS | 15.6 (12.6-23.7) | 18.7 (5.5-21.2) | NS | 17.8 (14.7-24.6) | 31.4 (13.8-62.1) | NS | 14.8 (8.5-21.5) | 38.9 (14.7-87.8) | **$p < 0.05$** | 13.6 (10.8-22.1) | 30.8 (22.0-42.1) | **$p < 0.05$** | 12.6 (8.8-12.9) | 17.4 (10.1-47.9) | NS |
| Total IgG anti-*Leishmania* (OD) | 78.5 (74-111) | 112.5 (95-205) | NS | 110.5 (74.2-143.0) | 97 (68.1-123-9) | NS | 241 (124-576.8) | 133 (108.4-221.6) | NS | 103.5 (95.2-118.5) | 399.5 (234-1376) | **$p < 0.05$** | 131 (101.8-112.8) | 817.5 (301.3-1939) | **$p < 0.05$** | 95.5 (89.4-171.0) | 1,933 (1,612-2,494) | **$p < 0.05$** |
| TCD4$^+$ levels (%) | - | - | NA | 45.2 (26.5-48.4) | 42.1 (40.9-49.8) | NS | 26.2 (19.9-31.3) | 27.4 (11.6-34.4) | NS | 32.2 (30.5-44.3) | 22.8 (15.4-40.6) | NS | 42.0 (34.8-47.3) | 36.5 (32.5-39.8) | NS | 44.1 (31.7-46.6) | 25.2 (20.7-27.3) | **$p < 0.05$** |
| CD25$^+$ in TCD4$^+$ levels (%) | - | - | NA | 3.9 (2.3-4.5) | 5.7 (2.2-19.9) | NS | 0.3 (0.1-2.8) | 14.0 (6.4-21.7) | **$p < 0.05$** | 11.7 (10.1-18.2) | 17.5 (7.8-46.5) | NS | 2.1 (0.7-12.9) | 18.3 (4.2-23.9) | **$p < 0.05$** | 0.3 (0.1-13.7) | 3.4 (2.5-19.7) | NS |

hpi: hours post-infection; dpi: days post-infection; g: grams; LPS: lipopolissacharide; NS: not significant; NA: not applicable; - : without data at this time; p-value by Mann-Whitney test. The results are expressed as median, IQR: interquartile range.

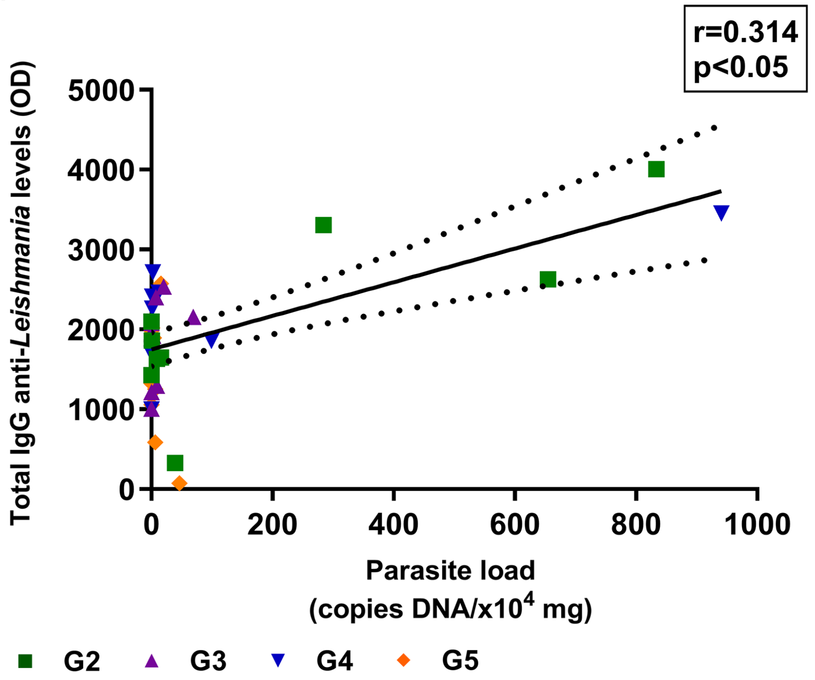


Fig. 1: correlations between parasite load and anti-*Leishmania infantum* IgG levels in the evaluated groups at 101-120 days post-infection (dpi). Positive correlation between anti-*L. infantum* IgG levels (OD) and parasite load (copies DNA/x$10^4$) (Spearman correlation, r = 0.31, $p < 0.05$). G2: infected and untreated (green square), G3, G4 and G5: infected and treated with antimonial (purple triangle), amikacin (blue inverted triangle) or both drugs (orange diamond), respectively.

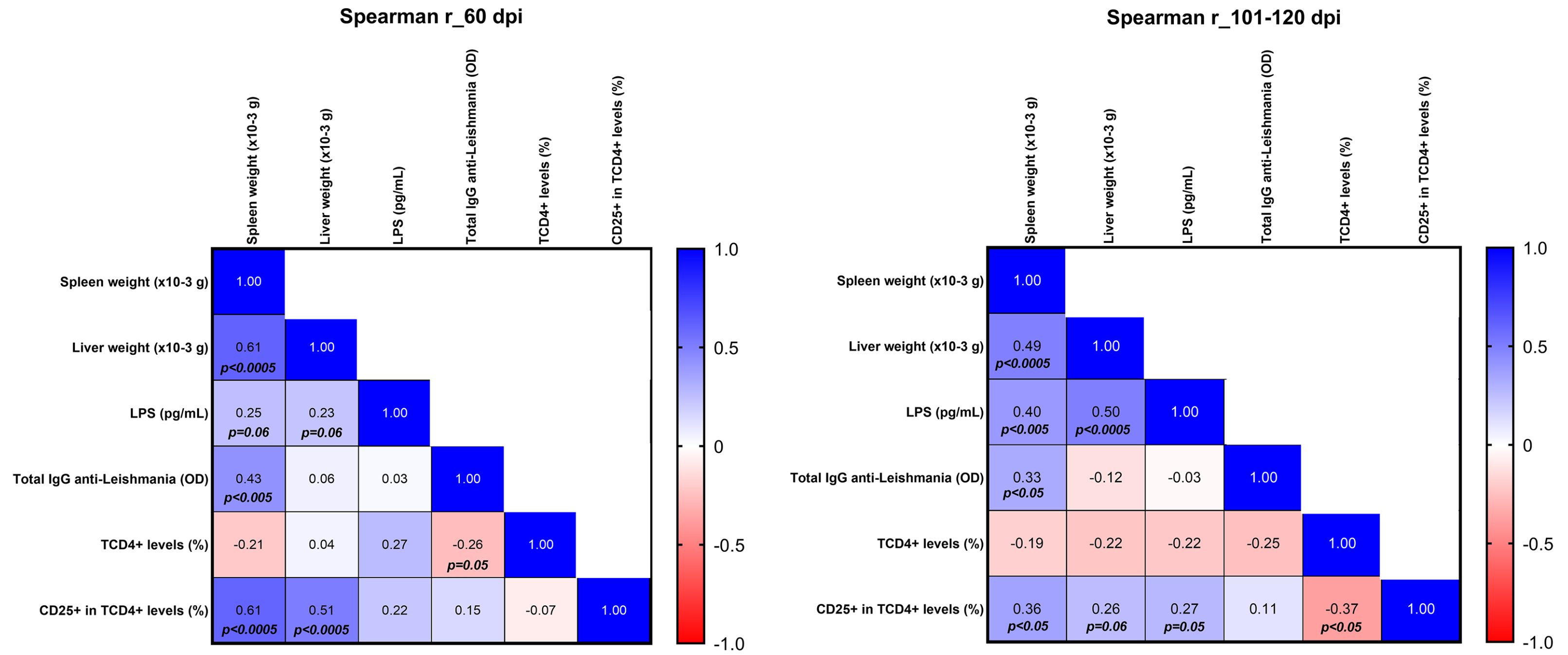


Fig. 2: correlation matrix between immunological and clinical parameters in the evaluated groups at 60 and 101-120 days post-infection (dpi). Correlation matrix showing the Spearman r and the *p* value of correlations between spleen weight, liver weight, LPS levels, anti-*Leishmania infantum* IgG levels (OD), $CD4^+$ T cell percentages and percentages of $CD25^+$ T cells at 60 and 101-120 dpi, including all groups evaluated: G1 (uninfected animal), G2 (infected and untreated), G3, G4 and G5 (infected and treated with antimonial, amikacin or both drugs, respectively). Red scale represents negative correlations and blue scale represents positive correlations.
